# Revealing the interplay between flow dynamics and cavitation activity in a flow-through sonoreactor

**DOI:** 10.1016/j.ultsonch.2026.107854

**Published:** 2026-04-15

**Authors:** Amirmohammad Javidani, Martine Poux, Joelle Aubin, Hélène Chaumat, Laurie Barthe

**Affiliations:** Université de Toulouse, Toulouse INP, CNRS, LGC, Toulouse, France

**Keywords:** Acoustic cavitation, Low-frequency ultrasound, Continuous sonoreactor, Sonochemiluminescence, Cavitation cloud, KI dosimetry

## Abstract

Acoustic cavitation is a well-known approach to intensify chemical and physical processes. Despite extensive studies in the understanding of batch ultrasonic reactors, flow-through sonoreactors, which are more relevant to continuous and large-scale operation, have received limited attention. Hence, in this study, the role of fluid flow rate and the relative direction between bulk flow and ultrasound propagation (co-current and counter-current) on sonochemical activity and cavitation cloud characteristics in a tubular flow-through sonoreactor (low frequency = 35 kHz) is systematically investigated under different ultrasonic amplitudes. Characterization techniques were employed, including calorimetry to quantify dissipated acoustic power and ultrasonic efficiency, shadowgraphy imaging to characterize the size of the cavitation cloud, sonochemiluminescence (SCL) to map chemically active cavitation zones, and KI dosimetry to quantify radicals. The results show that increasing flow rate slightly enhances calorimetric power, while ultrasonic efficiency remains largely unchanged. Shadowgraphy reveals that cavitation clouds elongate with increasing flow rate up to an intermediate value, beyond which the extent of the cavitation cloud decreases. In contrast, SCL mapping demonstrates that stagnant conditions and low flow rates are more favourable to induce larger and more intense chemically active cavitation zones, indicating that not all cavitation bubbles contribute equally to radical formation. The higher radical production at higher amplitudes and lower flow rates is shown through KI dosimetry. Moreover, counter-current operation enhances the distribution of chemically active cavitation and radical generation, as well as sonochemical efficiency when compared with co-current flow. This is attributed to modified hydrodynamics and increased residence time within the sonication zone.

## 1 Introduction

Over the last few decades, process intensification (PI) has emerged as a new strategy in chemical and process engineering, offering innovative solutions to enhance process efficiency while minimizing energy consumption and waste generation. These objectives can be achieved through the implementation and development of innovative reactor designs and equipment and/or the use of alternative energy sources [1]. Among the most promising PI approaches, acoustic cavitation (AC) has gained significant attention because of its outstanding feature to intensify both physical and chemical processes [2].

AC refers to the nucleation, expansion, and violent collapse of microbubbles when a liquid is subjected to ultrasound-induced pressure fluctuations [3]. Bubble collapse releases a great amount of energy, which subsequently raises the local temperature and pressure of the liquid up to approximately 5000 K and 1000 bar, respectively [4], [5]. These conditions promote the dissociation of water molecules into hydrogen radical (H ${\;}^{{}^{\circ}}$) and hydroxyl radical (${\;}^{{}^{\circ}}OH$), a highly reactive oxidizing agent that drives oxidation reactions [6]. In parallel, the formation of high-speed microjet streaming, intense shear forces, and shock waves during bubble collapse can intensify micro-mixing, local turbulence, and mass transfer [7]. Collectively, these phenomena are called cavitation effects, which are commonly classified into “chemical effects” (radical-mediated reactions) and “physical or mechanical effects” (hydrodynamic and transport enhancements). With this in mind, AC has found wide applications in environmental remediation (e.g., degradation of organic pollutants) [8], synthesis of chemicals [9], food processing [10], extraction processes [11], and fuel desulfurization [12].

Among the various sonoreactor configurations, the most widely used design employs an ultrasonic probe (horn) immersed in a reaction vessel [13]. Despite the maturity of batch ultrasonic reactors, continuous operation is generally preferred for implementation on a larger scale, owing to advantages like reduced processing time, lower waste generation and energy consumption, reduced labor cost, as well as allowing more compact process equipment [14], [15]. As a result, efforts have been made to transition from batch to continuous/flow-through sonoreactors. One of the simplest continuous designs involves equipping a conventional vessel/beaker with inlet and outlet ports on opposite sides, enabling continuous pumping of the feed solution into the reactor and simultaneous withdrawal of the sonicated solution [16]. This configuration can be operated as a single stage [17] or as multi-stage reactors in series [18], [19], [20]. Alternatively, the ultrasonic probe may be inserted into a flow cell, which has shown promising for pilot-scale implementation [21]. More advanced designs incorporate multiple ultrasonic transducers or horns [22], either clamped onto a tubular reactor [23], [24] or mounted on vessel walls in various arrangements [25].

When flow sonoreactors are applied in chemical and physical processes, the flow rate has been widely reported as a key operating parameter. According to literature, the external flow can influence the cavitation performance through modifying i) residence time within the sonication zone, ii) cavitation bubble dynamics, shape, and collapse intensity, and iii) hydrodynamic behavior and mixing inside the sonoreactor.

Residence time in the active sonication region is often the dominant factor governing performance in flow sonoreactors. Lower flow rates increase the exposure time of the liquid to the acoustic field, enhancing interactions between cavitation-generated radicals and target compounds [26]. Higher desulfurization efficiencies at lower flow rates have been attributed to longer residence time in the sonoreactor [27]. Similar findings have also been reported in other studies on sonochemical degradation of chlorinated hydrocarbon [28], oxidative desulfurization of diesel [29], and biodiesel production [30]. Gondrexon et al. [19] attributed the adverse effect of increasing flow rate on pentachlorophenol degradation to the insufficient contact time between the pollutant and the reactive radicals. Similarly, Juretic et al. [31] observed that the flow rate is inversely proportional to the amount of produced radicals. Hulsmans et al. [32] also reported lower disinfection rates at higher flow rates in single-pass mode. It should be noted that, in the above-mentioned studies, although lower flow rates were found to be more favorable, they inevitably lead to a reduced overall production capacity in continuous systems.

External bulk fluid flow can also influence bubble collapse dynamics by enhancing bubble transport and deformation, often promoting non-spherical collapses rather than spherical collapses. Such asymmetric collapses have been shown to reduce hotspot temperatures and pressures in single bubble– sonoluminescence measurements [33]. Recently, Wang and coworkers [34] demonstrated that, when a cavitation bubble is exposed to an external flow, the bubble size, its lifespan, as well as its deformation are notably influenced. Calvisi et al. [35] showed that non-spherical collapses of bubbles led to lower collapse pressures and temperatures than for spherical ones, and this effect becomes more pronounced at higher acoustic pressures. Thus, it can be said that flow-induced shape instabilities might weaken the hotspot condition. Wood et al. [36] reported that the imposed flow can inhibit bubble coalescence, thereby reducing cavitation activity. In another paper, the authors mentioned that at high frequency, the external flow had a minor influence on sonochemiluminescence (SCL) intensity and the overall yield of generated radicals. They also highlighted the importance of bubble transience, collapse time, and fragmentation dynamics under flow mode [37]. Gielen et al. [38] postulated that fluid flow can favor the formation of stable cavitation by reducing the average bubble size.

The hydrodynamic behaviour and mixing performance in sonoreactors are also influenced by an external flow rate. Parvizian et al. [39] showed that a lower flow rate is more favorable in terms of micromixing performance. When an external flow rate is applied to a sonoreactor, it is important to understand the interaction between acoustic streaming and bulk flow, which determines the overall hydrodynamics of the system and where cavitation occurs. Favorable flow fields can redistribute cavitation into inactive regions or prevent bubble accumulation in acoustically shielded zones, thereby enhancing sonochemical performance. In this regard, Fattahi et al. [40] reported that at low ultrasonic power, external flow strongly interacts with the acoustic jet, disrupts the streaming pattern, thereby prolonging residence time in the active zone and improving sonochemical activity, whereas its influence becomes negligible at higher power levels. Valitov et al. [41] demonstrated that variations in flow rate modify the interaction between the fluid flow and the acoustic field. This may lead to the formation of vortices and alter the flow streamlines, thereby changing the residence time distribution and affecting crystallization performance. In a novel flow sonoreactor, in which the ultrasonic probe was attached perpendicularly to a rectangular duct, the flow was observed to deflect and reshape the acoustic streaming-induced plume [42]. Most of the aforementioned studies have examined the effect of flow rate in homogeneous liquid systems. However, in practical applications, cavitation processes often occur in multiphase environments, where the presence of suspended solids, droplets, or gas phases may modify the interaction between fluid flow and cavitation. For example, the presence of solid in the medium has been reported to cause ultrasound attenuation [43], while the solid particles may also act as additional nucleation sites for cavitation bubbles [44].

In several investigations, it has been reported that an optimal flow rate needs to be selected to maximize the cavitation performance. For example, Momin and Gogate [45] stated that, within the recirculation range of 200–800 ml/min, the flow rate at 400 ml/min gave the highest extent of decolorization. Another study suggested that, depending on the applied frequency and power, an optimal flow rate must be chosen to maximise the SCL intensity [46]. Ajie et al. [47] observed a significant decrease in radical production above a certain flow rate. Sidnell et al. [48] reported that moderate recirculating flow rates enhanced PFAS defluorination by about 14%, whereas higher rates brought the rate back to values comparable to the no-flow case, likely due to flow-induced quenching of bubble collapse temperature and suppressing radical generation. Notably, the optimum flow rate was also observed to depend on both US power and probe geometry [40].

Although the influence of fluid flow rate on cavitation performance has been widely investigated, the majority of existing studies have been conducted in beaker- or vessel-based sonoreactors in flow-through or recirculating operations. However, such reactors do not truly represent continuous processing conditions, since broad residence time distribution and the presence of dead zones with an absence of cavitation allow part of the liquid to bypass the cavitation zones or be exposed to ultrasound for a short time. To overcome these limitations and move toward real-world continuous implementation, confined flow geometries, such as tubular sonoreactors, are a more suitable choice. Despite their practical relevance, the fundamental interplay between fluid flow and cavitation activity remains insufficiently understood in the current literature and it remains unclear to what extent conclusions drawn from vessel-based studies can be applied to confined continuous sono-systems. In addition, insights into how the flow rate and its direction relative to ultrasonic wave propagation (co-current or counter-current configurations) influence cavitation behavior and sonochemical performance are still lacking. Thus, the present work introduces a novel tubular flow-through sonoreactor design, operating at low frequency (35 kHz), and provides a systematic investigation of the coupled effects of flow rate, flow direction, and ultrasonic amplitudes on sonochemical activity and cavitation cloud characteristics in a homogenous system. To this end, a set of characterization techniques, including calorimetric measurements, shadowgraphy imaging, sonochemiluminescence (SCL), and KI dosimetry, are employed under different operating conditions.

## 2 Materials and method

### 2.1 Chemicals

Potassium iodide (99%), sulfuric acid (1 M), and sodium hydroxide (>97%) were supplied by Fisher Scientific, and luminol (97%) was purchased from Sigma Aldrich. All the chemicals were utilized without further purification. The required quantities of chemicals were weighed using an analytical balance (Sartorius Practum 224-1S), and aqueous solutions were prepared with distilled water at ambient temperature.

### 2.2 Experimental setup

**Fig. 1:**
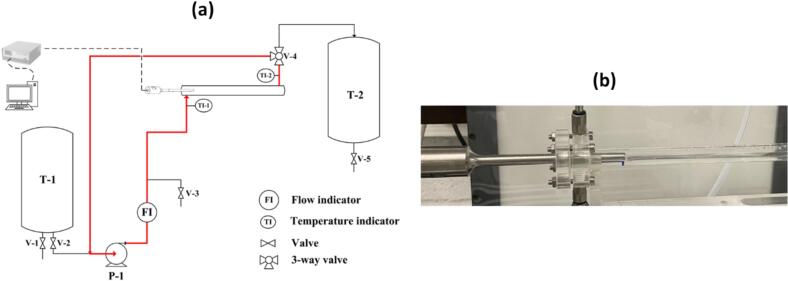
(a) Schematic diagram of the experimental setup, and (b) tubular flow-through sonoreactor.

A schematic diagram of the experimental setup is depicted in Fig. 1(a). It consists of two 10-liter tanks: Tank 1 (T-1) as the feed reservoir, and Tank 2 (T-2) as the drain tank. The liquid was pumped from the feed tank to the tubular sonoreactor using a magnetically coupled gear pump (D series pump, Tuthill, Netherlands) (P-1), coupled to a flow meter (mini CORI-FLOW M15, Bronkhorst, Netherlands). The flow rate (FI) was adjusted between 200 and 1800 ml/min, corresponding to a residence time in the tube of H° 0.8–7.1 s, by modifying the pump rotation speed. Two PT-100 temperature sensors were installed at the inlet and outlet of the tubular reactor (TI-1 and TI-2) for monitoring of ΔT. The sensors had an accuracy of ± 0.03 °C and a resolution of 0.01 °C (France Thermomètre). The valve installed in the downstream of the tubular reactor (V-4) allowed operation either in single-pass continuous mode or recirculating (closed-loop) mode, as indicated by the red flow path in Fig. 1(a). Also, the sampling port (V-3) allows sample collection and drainage of the remaining water in the tubing after washing. By interchanging the inlet and outlet connections of the tube, the direction of liquid flow relative to ultrasound propagation could be configured in either co-current or counter-current mode.

The sonoreactor consists of a transparent PMMA tube with an inner diameter of 1 cm, a wall thickness of 0.2 cm, and a length of 30 cm (Fig. 1(b)). A truncated-cone ultrasonic probe (22D6, low frequency of 35 kHz) with a tip diameter of 0.6 cm was inserted horizontally into the tube, penetrating 3.9 cm and positioned at the center of the tube cross-section. This insertion depth was determined based on the nodal position along the sonotrode body, where the vibration amplitude is minimal. Locating the connection at this nodal point ensures mechanical stability and prevents excessive vibration at the junction between the probe and the tubular reactor. Moreover, the relatively high probe-to-tube diameter ratio was intentionally selected to limit the possibility of the fluid bypassing the cavitation zone.

The ultrasonic transducer was driven by a computer-controlled ultrasonic generator (NexTgen Inside 500), which enabled adjustment of the ultrasonic amplitude as well as real-time monitoring and recording of input electrical power during ultrasonication. The ultrasonic system was supplied by SinapTec (Lezennes, France).

### 2.3 Calorimetric measurements and US efficiency

Calorimetry was employed to determine the effective ultrasonic power delivered into the liquid. This method is based on the assumption that, under steady operating conditions, the ultrasonic energy dissipated into the liquid is primarily converted into heat, resulting in an increase in the liquid temperature [49]. Based on this temperature rise, calorimetric power ($P_{\mathrm{cal}}$) can be obtained. The calorimetry experiments were performed using distilled water in single-pass continuous mode. All experiments were carried out in triplicate, and the reported results correspond to mean values with their associated standard deviations.

For each operating condition, the ultrasonic efficiency (η), defined as the fraction of the net electrical power ($P_{\mathrm{elec}}$) converted into acoustic power within the liquid, was calculated as follows [50]:

(1) $$\eta \left(\%\right)=\frac{P_{\mathrm{cal}}}{P_{\mathrm{elec}}}\times 100$$

The electrical power was obtained as the time-averaged electrical power over 2 min of continuous sonication, excluding the initial 2 s (duration to have stabilized transducer power).

### 2.4 Shadowgraphy imaging

The acoustic cavitation cloud field was visualized via shadowgraphy imaging, a technique based on differences in light refraction and scattering between the cavitation vapor phase and the surrounding liquid. This technique enables the visualization and localization of cavitation cloud zones (vapor and/or gas-filled bubbles) within the reactor. For the purpose of characterizing time-averaged cavitation cloud features, such as cloud localization, shape, and area, a digital camera (U3-3160CP-C-HQ, IDS, Germany) was used in this study. It was operated at a frame rate of 25 frames per second (fps), with a resolution of 25 μm/pixel and an exposure time of 8 ms. Backlighting was provided by a rectangular LED panel supplying continuous illumination and placed on the opposite side of the camera. All shadowgraphy experiments were done using distilled water at ambient temperature (without dissolving any gases) in continuous mode. Due to the oscillatory and transient nature of cavitation, time-averaged images were generated for each operating condition by averaging over 500 consecutive 2D raw frames, thereby representing the mean cavitation cloud structure. The averaged images were subsequently calibrated through subtracting the raw image from the reference image (silent mode) and converted to an 8-bit format for post-processing in MATLAB. More details on the shadowgraphy imaging technique can be found in the Supplementary Materials.

### 2.5 Sonochemiluminescence (SCL) mapping

The SCL mapping was performed under the same operating conditions as the shadowgraphy experiments. Unlike shadowgraphy, which visualizes the overall cavitation cloud structure, SCL images enable mapping and spatial distribution of radical-producing bubbles.

SCL technique is based on the reaction of luminol with AC-generated oxidants (mainly ${\;}^{{}^{\circ}}OH$) under alkaline conditions. This reaction leads to the formation of excited-state 3-aminophtalatae (${3-APA}^{*}$), which then relaxes to its ground state by emitting blue light (wavelength close to 430 nm) [51]. Since this emission is directly driven by hydroxyl radicals produced during acoustic cavitation, the bright zones in SCL images correspond to cavitation chemically active (radical-producing) zones, and the intensity of emitted light is believed to reflect the local cavitation activity, which depends on both the number of active bubbles and their collapse intensity [52].

In our experiments, a luminol solution was prepared by dissolving 0.1 g/L of luminol in 1 g/L of sodium hydroxide (NaOH), resulting in a solution with pH ≈ 12.5. All the SCL imaging were performed in single-pass continuous mode. To avoid interference from external light, the SCL imaging was undertaken in a dark room. SCL images were acquired using a digital exposure-controlled camera (Sony α7 III) equipped with a 50 mm focal length lens, operated at ISO 5000, an exposure time of 30 s, and an aperture of *f*/7.1. The camera was also positioned 60 cm from the tubular sonoreactor, and the focus was adjusted at the sonotrode tip. Image acquisition was initiated approximately 2 s after the onset of ultrasonication. The resulting images had a spatial resolution of 40 μm/pixel. To eliminate background noises, all SCL images were calibrated by subtracting from the reference image, which was acquired under silent (non-sonicated) mode. Then, the resulting RGB images were converted to 8-bit images, and they were processed to determine SCL intensity and area. Details on the calculation of the total SCL intensity are provided in the Supplementary Materials.

### 2.6 Potassium iodide dosimetry and sonochemical efficiency

Potassium iodide (KI) dosimetry is a well-established experimental method for indirectly measuring the total generation of hydroxyl radicals. Although this method has certain limitations compared with other chemical dosimetry techniques, it remains a widely used and reliable approach for evaluating sonochemical activity [53]. The extreme conditions during bubble collapse lead to the dissociation of water molecules to form highly reactive hydroxyl radicals (${\;}^{{}^{\circ}}OH$). These radicals subsequently react with iodide ions ($I^{-}$), resulting in the liberation of iodine ($I_{2}^{-}$), which further reacts with excess $I^{-}$ to ultimately generate triiodide ions ($I_{3}^{-}$) [54]. So, the concentration of $I_{3}^{-}$ ($C_{I_{3}^{-}}$) is an indicator of the quantity of hydroxyl radicals produced.

In the present study, an aqueous KI solution with a concentration of 10 g/L was prepared. To suppress undesired side reactions, it has been suggested to acidify the medium [55], [56]. So, the solution was acidified to pH = 4 using sulfuric acid. The solution was stirred using a magnetic stirrer at 500 rpm for 20 min to ensure complete dissolution of KI. Prior to each experiment, the reactor and all tubing were thoroughly rinsed with tap water. The KI dosimetry experiments were conducted under closed-loop (recirculating) mode. After setting and stabilizing the desired flow rate, ultrasonication was applied for 5 min. After that, three samples ( 5 ml each) were withdrawn from the sampling port (V-3). Afterwards, the absorbance was measured using a UV–Vis spectrophotometer (Shimadzu UV mini 1240) at a wavelength (λ) of 353 nm. After calibration, the $I_{3}^{-}$ concentration was then calculated using the Beer–Lambert law (molar absorptivity of $I_{3}^{-}$ = 26303 L/mol.cm) [57]. The experiments were performed in triplicate, and the concentration for each experiment corresponds to the average value obtained from the three collected samples.

In sonochemical reactors, it is also crucial to evaluate the energy efficiency of the radical production, which is termed as sonochemical efficiency (*SE*) (µmol/J) [58], and was calculated as:

(2) $$\mathrm{SE}=\frac{C_{I_{3}^{-}}\times V_{\mathrm{loop}}}{P_{\mathrm{cal}}\times t}$$

where, $V_{\mathrm{loop}}$ denotes the volume of the closed loop (140 ml), and t is the sonication time (5 min).

## 3 Results and discussion

### 3.1 Calorimetric power and US efficiency

To investigate the effects of flow rate and US amplitude on calorimetric power and US efficiency, the measurements in co-current mode were conducted at flow rates of 200, 600, 1000, 1400, and 1800 ml/min, corresponding to Reynolds numbers in the tube of 423, 1270, 2118, 2965, and 3812, respectively. In our ultrasonic system, an amplitude of 40% is the minimum threshold required to trigger cavitation. Hence, in all experiments, the US amplitude was set within the range of 40–100%. For the calorimetry in counter-current mode, the same flow rates as those used in the co-current configuration were investigated at amplitudes of 60% and 100%.

#### 3.1.1 Co-current configuration

**Fig. 2:**
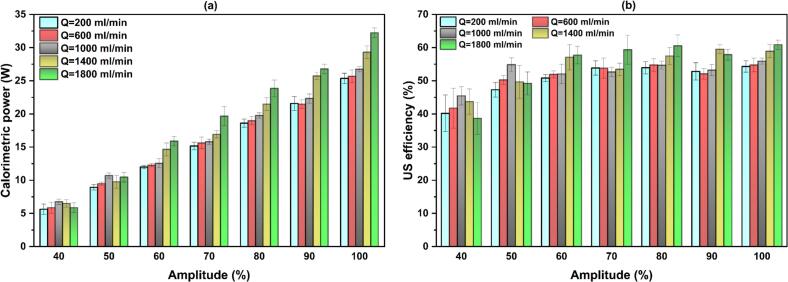
(a) calorimetric power and (b) US efficiency at different operating flow rates in co-current configuration.

Fig. 2(a) compares the calorimetric power obtained at varying flow rates for each US amplitude. A linear increase in calorimetric power with increasing amplitude is observed, regardless of the applied flow rate, indicating effective transfer of electrical energy into acoustic energy within the ultrasonic system. This confirms the proper functioning of the power supply US transducer–tubular reactor assembly, with no evidence of abnormal acoustic losses. It can also be observed that at low amplitudes (A = 40 and 50%), the calorimetric power is weakly influenced by the flow rate, which might be due the fact that cavitation is close to its inception threshold. Yet, at higher amplitudes (A ≥ 60%), a slight and gradual increase in calorimetric power with increasing the flow rate is observed, with the most enhancement at Q = 1800 ml/min. This behavior shows that, once a more developed cavitation is established, increased flow promotes more effective dissipation of acoustic energy into the working fluid, likely owing to improved heat transport and interaction between the cavitation and fresh liquid entering to the tubular sonoreactor.

The impact of flow rate on the electrical power is illustrated in Fig. S1(a). For all the set amplitudes, raising the flow rate results in a higher electrical power demand, and this becomes more pronounced at higher amplitudes. Consequently, additional electrical energy must be supplied to the transducer to maintain a fixed vibration amplitude under higher flow rates. Since the ultrasonic efficiency is the ratio of calorimetric power to electrical power, the corresponding US efficiency shows a weak dependence to flow rate, as shown in Fig. 2(b). According to this figure, the minimum efficiency observed at A = 40% is likely associated with operation near the cavitation onset threshold, where cavitation intensity is weak and acoustic energy dissipated into the liquid is limited. However, amplitudes of higher than 50% results in a relatively stable energy conversion efficiency within the range of 47–60%. An efficient sonoreactor is expected to exhibit not only high but also stable ultrasonic efficiency across varying operating conditions, which is observed at A ≥ 50% in the present sonoreactor.

#### 3.1.2 Counter-current configuration

**Fig. 3:**
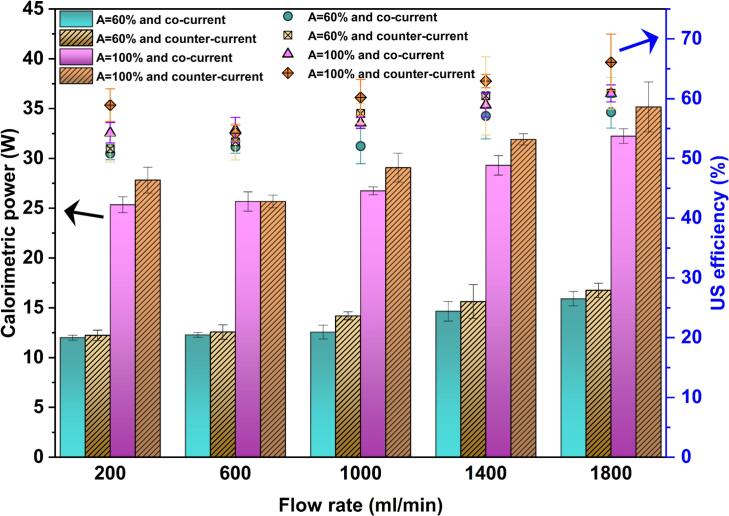
Comparison of calorimetric power and US efficiency between co-current and counter-current modes.

Fig. 3 compares the calorimetry power and US efficiency under co-current and counter-current operations. As shown, counter-current mode results in a slightly higher calorimetric power than in co-current mode at both set amplitudes. Meanwhile, a modest increase in electrical power is observed under counter-current conditions (Fig. S1(b)) because of higher hydrodynamic resistance to probe vibration. However, since the increase in calorimetric power is slightly more dominated than the corresponding increase in electrical power, the resulting US efficiency is marginally higher in counter-current compared to its co-current counterpart.

### 3.2 Cavitation cloud characteristics using shadowgraphy

#### 3.2.1 Co-current configuration

**Fig. 4:**
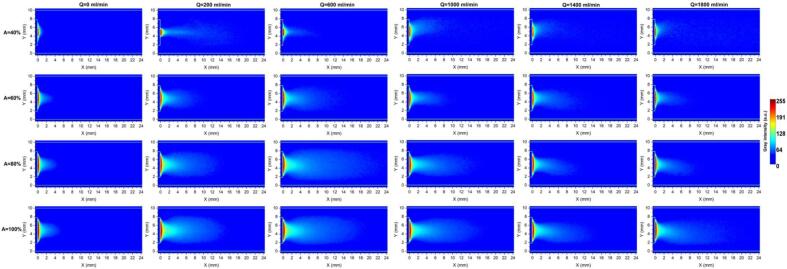
Colormap shadowgraphy images obtained under various flow rates (0 – 1800 ml/min) and amplitudes (40% – 100%) in co-current mode.

To characterize the cavitation cloud in co-current operation, shadowgraphy imaging was conducted under both no-flow mode (Q = 0) and flow conditions (Q = 200, 600, 1000, 1400, and 1800 ml/min) at four different amplitudes (40, 60, 80, and 100%). Fig. 4 presents the colormap images from shadowgraphy acquired downstream of the sonotrode tip in co-current mode. Under all the studied operating conditions, a large conical cavitation cloud structure is observed in a confined zone adjacent to and attached to the sonotrode tip (primary cavitation zone). This zone exhibits high local cavitation concentration and structural coherence. Notably, this dense cloud maintains a symmetrical cone-like shape regardless of the operating flow rate and amplitude, indicating that dense cloud morphology is mainly determined by acoustic energy rather than convective hydrodynamic effects. Similar conical morphology have also been observed for pure water in previous studies [59], [60], [61], [62]. The accumulation of this highly dense cloud near the sonotrode tip is mainly governed by primary Bjerknes forces, which drive the bubbles toward the pressure antinodes of the acoustic field existing in the proximity of sonotrode surface. Apart from that, secondary Bjerknes forces arising from bubble–bubble interactions promote their attraction, leading to attached cavitation clusters and enhanced cloud coherence [59], [60].

**Fig. 5:**
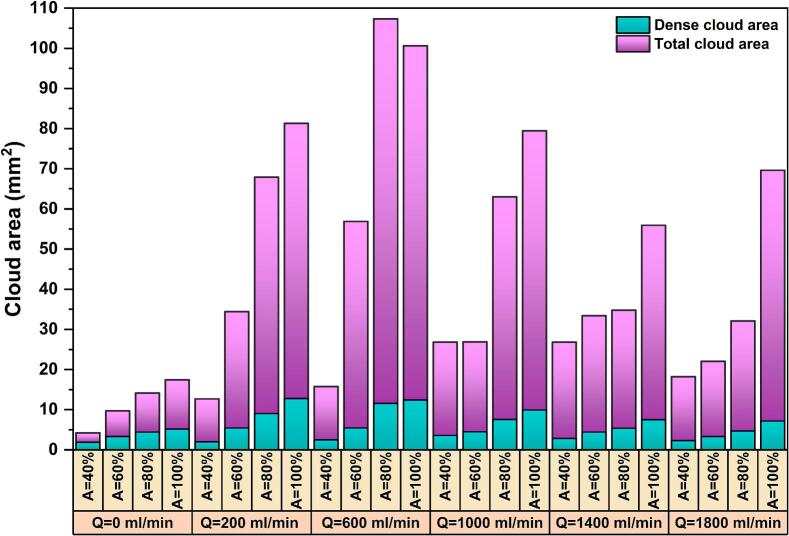
Cloud area (dense and total zones) under various flow rates and amplitudes in co-current condition.

The variation of the dense cavitation cloud area under different flow rates and amplitudes is illustrated by the cyan bars in Fig. 5. A linear relationship between the dense cloud area and ultrasonic amplitude is observed under no-flow (Q = 0) and low-flow (Q = 200 ml/min) conditions. Notably, at Q = 200 ml/min, the dense cloud area exhibits a higher sensitivity to amplitude compared to the no-flow case. A similar linear dependence between cavitation cloud area and calorimetric power was recently reported by Viciconte et al. [63]. However, at higher flow rates (≥ 600 ml/min), this linear trend is slightly disrupted, which shows that increased bulk flow modifies the development of dense zone with increasing amplitude. Similar observations have been reported when other operating parameters, such as ambient pressure, were varied [64].

Looking at the impact of flow rate on the dense cloud area reveals a non-monotonic trend across all amplitudes. The dense area initially increases with increasing flow rate, reaches a maximum, and subsequently decreases at higher flow rates. At low flow rates, the external fluid flow promotes the development of the cavitation dense zone by continuously renewing cavitation nuclei and assisting acoustic streaming in redistributing bubbles in front of the sonotrode tip. As a consequence, the cavitation bubbles are less likely to accumulate within a highly confined zone. Beyond a critical flow rate (depending on the set amplitude); however, further increases in flow rate lead to a reduction in the dense cloud area. This behavior can be attributed to the fact that a strong external flow can suppress bubble coalescence and sweep the bubbles out of the intense acoustic field faster than they can grow and cluster. As a consequence, the residence time of the bubbles within this compact zone may become insufficient to create a coherent and compact cloud. It is worth noting that a larger and denser cavitation zone does not necessarily yield higher cavitation performance, as the highly dense bubbles associated with high void fraction in a small zone may attenuate ultrasound wave propagation in the reactor [65].

From the images in Fig. 4 in addition to the dense primary cavitation cloud attached to the sonotrode tip, the shadowgraphy images unveil the presence of a secondary cavitation cloud extending downstream. This appears as a mist-like cavitation field or discrete bubble clusters, which corresponds to a gray-scale intensity of 40 < *I* < 80. The secondary cavitation cloud originates from bubbles detached from the primary cloud and transported downstream mainly by acoustic streaming. In this zone, Bjerknes forces are weaker, and as a result, bubbles are less likely to interact and coalesce, leading to a more spatially distributed and less coherent cavitation structure than the primary cloud. The cavitation in this zone can appear as isolated spherical bubbles [63], [64]. Focusing on the secondary cavitation cloud at A = 40%, the structure remains largely symmetrical up to a flow rate of 600 ml/min. At higher flow rates (Q ≥ 600 ml/min), the mist-like cloud bends toward the upper wall of the tube, which is probably due to the position of the fluid inlet, which is perpendicular to the main tube upstream of the sonotrode. Under these conditions, the imposed flow can govern the spatial distribution of the secondary cavitation cloud. In contrast, when a higher amplitude is set (A = 60, 80, and 100%), the secondary cloud largely preserves its symmetrical structure across all the flow rates, showing that stronger acoustic irradiations mitigate the bulk flow-induced asymmetry.

The total cavitation cloud area (including both the dense primary cloud and the secondary mist-like cavitation) is also shown in Fig. 5. Similar to the results of the dense area, under all the US amplitudes, the total area initially increases with flow up to a point, and then decreases at higher flow rates. At A = 40%, the largest cloud area is observed at Q = 1000 and 1400 ml/min, whereas for higher amplitudes (A = 60–100%), the maximum area occurs at Q = 600 ml/min. At higher flow rates, the decreasing trend is associated with minor fluctuations for A = 60 and 100%, which might be linked to the competing effects of bulk flow and acoustically driven bubble transport.

**Fig. 6:**
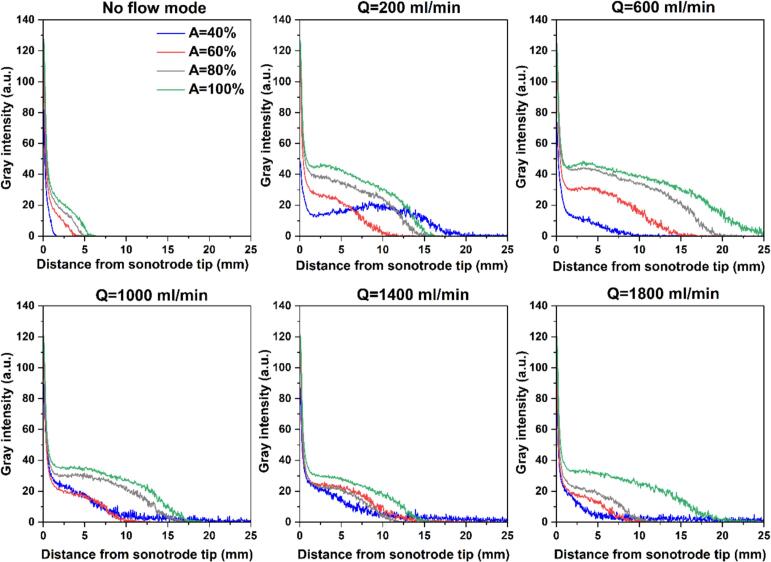
Gray intensity profiles along the tube length (each data point representing diameter-averaged intensity) at different amplitudes and flow rates in co-current mode.

To further quantify the spatial development of cavitation under co-current mode, intensity profiles were obtained along the tube length, with each data point representing the average intensity over the tube diameter. This analysis gives information about the axial extent of cavitation, which is useful for estimating the effective length or volume required in a continuous tubular sonoreactor. According to Fig. 6, all profiles start at a maximum intensity near the sonotrode tip, which reflects the high local bubble density. This is followed by a sharp initial decay within the first ∼ 2 mm downstream of the tip, attributed to steep cavitation concentration gradients, after which a more gradual decay is observed until the intensity approaches zero, marking the effective downstream limit of the bubbly zone. From Fig. 6, it is also evident that under all the studied flow rates, raising the US amplitude from 40% to 100% leads to a clear elongation of the intensity profile and a delayed decay of cavitation along the axial direction, thereby shifting the vanishing point forward. This indicates that higher amplitudes sustain cavitation in the far-field zones, mainly due to a larger bubble population near the sonotrode as well as stronger acoustic streaming and bubble shedding. A notable exception occurs at Q = 200 ml/min and A = 40% (close to the cavitation onset power), where cavitation forms a narrow jet-like plume that extends farther downstream (clearly seen in Fig. 4). In addition, at flow rates above 1000 ml/min, the decay curves corresponding to 40–80% amplitude become very similar, likely due to their disruption by the dominating bulk flow.

Looking at the influence of flow rate for each given amplitude from Fig. 6, it is found that the no-flow condition (Q = 0 ml/min) exhibits the fastest decay of intensity, indicating that cavitation is highly localized near the sonotrode tip surface in stagnant fluid. The introduction of flow generally slows the axial decay, reflecting enhanced downstream transport and persistence of cavitation bubbles as discussed earlier. For US amplitudes between 60% and 100%, increasing the flow rate up to 600 ml/min results in a progressively slower decay of intensity, and Q = 600 ml/min shows the most extended cavitation events. However, further increases in flow rate lead to a faster decay and earlier disappearance of the intensity profiles. At an amplitude of 40%, a different trend is observed. The most sustained intensity occurs at Q = 200 ml/min, while further increases in flow rate yield similar vanishing points. Overall, these trends closely reflect the behavior observed for cavitation cloud area, and it can be said that a modest flow rate is more favorable for maximizing the spatial extent of cavitation.

#### 3.2.2 Counter-current configuration

**Fig. 7:**
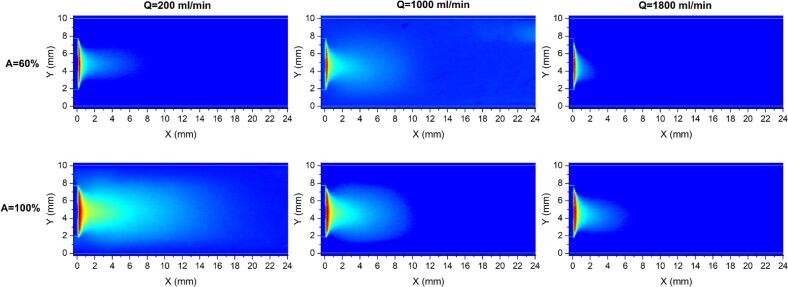
Shadowgraphy images in counter-current mode under flow rates of 200, 1000, and 1800 ml/min, and US amplitudes of 60 and 100%.

**Fig. 8:**
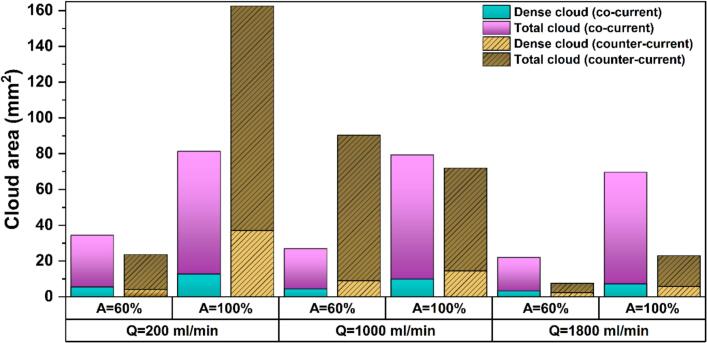
Comparison of dense and total cloud area for the two configurations (counter-current and co-current) at different flow rates and amplitudes.

**Fig. 9:**
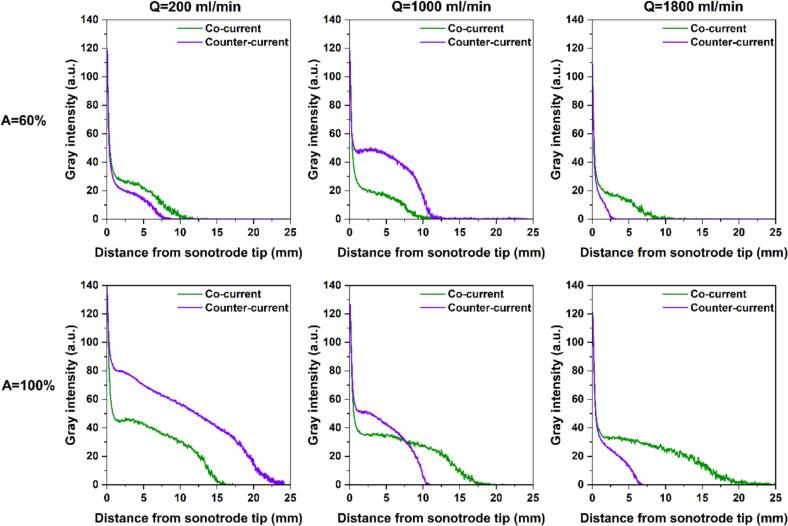
Comparison of gray intensity profiles along the tube length (each data point representing the diameter-averaged intensity) between co-current and counter-current configurations at Q = 200, 1000, and 1800 ml/min and A = 60 and 100%.

Fig. 7 presents the shadowgraphy images in counter-current mode for three flow rates (Q = 200, 1000, and 1800 ml/min) and amplitudes of 60 and 100%. Also, comparisons of dense and total cloud area as well as gray intensity profiles between co-current and counter-current are given in Fig. 8, Fig. 9, respectively. Reversing the flow direction in the sonoreactor alters the cavitation cloud field differently depending on the set operating parameters. When the US amplitude was set at 60%, counter-current mode results in a slightly faster axial attenuation of gray intensity at Q = 200 ml/min, which is consistent with the smaller dense and total cloud area when compared with co-current flow. In contrast, at a flow rate of 1000 ml/min, counter-current operation produces a more extended intensity profile and larger cavitation cloud areas than co-current flow. At a higher flow rate of 1800 ml/min, counter-current operation again becomes less favorable, leading to reduced cloud areas and an upstream shift of the vanishing point, likely due to dominant hydrodynamic effects that disrupt both bubble clustering and acoustic streaming, and shorten residence time within the acoustic field.

Under a US amplitude of 100%, counter-current mode at 200 ml/min exhibits a delayed decay of gray intensity and larger dense and total cavitation cloud areas compared with co-current flow, indicating enhanced cavitation persistence under low-flow conditions. At Q = 1000 ml/min, counter-current operation yields a higher near-field gray intensity close to the sonotrode tip, consistent with the increased dense cloud area; however, the axial intensity profile decays more rapidly in the far-field region compared with co-current flow, and the total cloud area is slightly reduced. At Q = 1800 ml/min, counter-current significantly reduces the total cloud area and shifts the cavitation vanishing point upstream, indicating that strong opposing flow suppresses downstream cavitation persistence even at high ultrasonic amplitude.

### 3.3 Sonochemiluminescence (SCL) mapping

#### 3.3.1 Co-current configuration

**Fig. 10:**
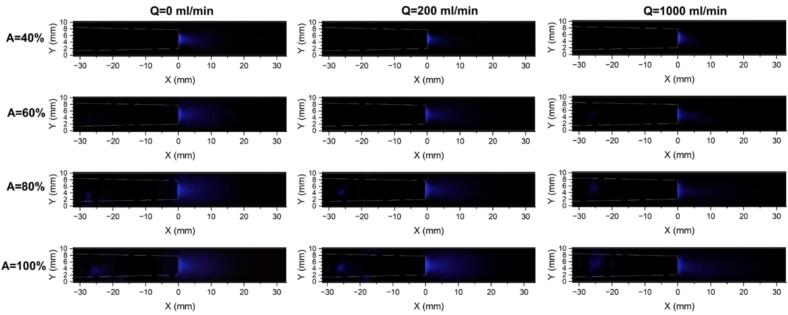
Sonochemiluminescence (SCL) images obtained under stagnant fluid, Q = 200 and 1000 ml/min operating under co-current mode, and different US amplitudes from 40 to 100%.

**Fig. 11:**
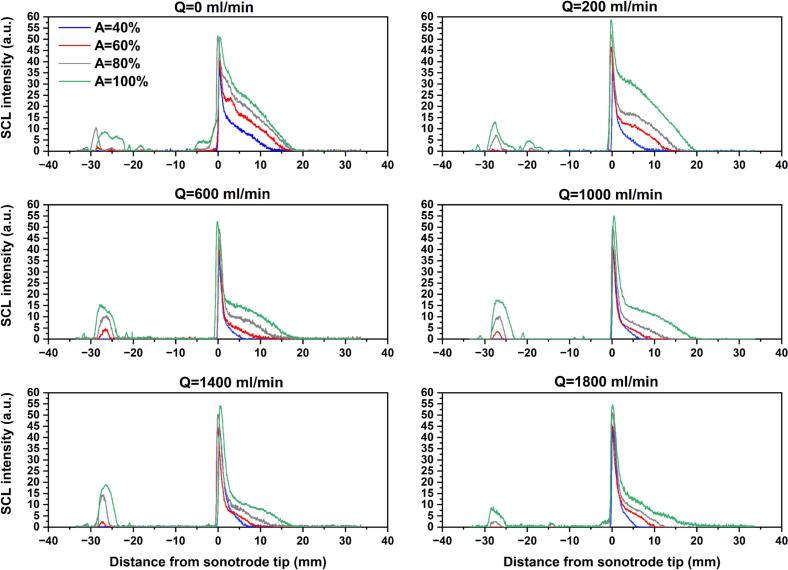
Effect of US amplitude on the SCL intensity along the length of the tube in co-current mode for flow rates ranging from 0 to 1800 ml/min.

Fig. 10 shows the SCL images obtained under no-flow mode and at flow rates of 200 and 1000 ml/min for four US amplitudes under co-current operation, and Fig. S2 also presents the images at flow rates of Q = 600, 1400, and 1800 ml/min. To quantify and compare the effects of flow rate and US amplitude, the SCL intensity was extracted along the defined ROI, with the sonotrode tip as the reference position (X = 0). The SCL intensity was calculated along the length of tube, where each data point corresponding to the diameter-averaged intensity, and the resulting profiles are presented in Fig. 11. It is clear that, for all scenarios, a sharp peak in SCL intensity is observed at the sonotrode tip, indicating strong chemical activity in this region. This behavior is consistent with the shadowgraphy results and shows that the large number of bubbles accumulated in this zone are chemically active. Similar behavior in a batch US reactor has been reported in previous studies [52], [56]. Downstream of the tip, the SCL intensity gradually decreases, marking the attenuation of chemical activity with distance. However, the decay behavior is highly influenced by the imposed value of US amplitude. Increasing the amplitude from 40% to 100% gives rise to a remarkable growth of the SCL intensity zone, showing that higher amplitudes sustain radical-producing bubbles in a larger axial distance. At higher amplitudes, a small SCL peak is also observed upstream of the sonotrode tip (around 30 mm), owing to the appearance of lateral ultrasonication.

**Fig. 12:**
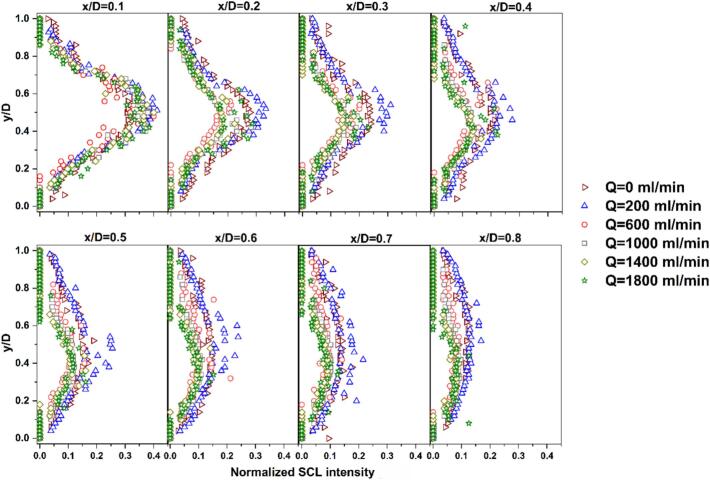
Normalized SCL intensity at different normalized distances from sonotrode tip (x/D) for varying flow rates and amplitude of 100% working under co-current flow.

By comparing profiles of identical colors in Fig. 11, the effect of flow rate on the SCL intensity can be assessed. At A = 40% (blue profiles), the no-flow mode is observed to have the longest downstream penetration of SCL intensity, while all flow conditions show similar and more rapidly decaying profiles. For amplitudes of 60% and 80%, the no-flow case again displays the most extended reaction zone, followed by the low flow rate of 200 ml/min, and higher flow rates lead to similar decay trends as well as earlier disappearance of SCL intensity. When the amplitude is increased to 100%, the SCL penetration at Q = 200 ml/min slightly exceeds that of Q = 0 ml/min, and they are significantly larger than those obtained at higher flow rates (Q ≥ 600 ml/min). These trends are further confirmed by plotting the normalized SCL intensity (ratio of SCL intensity to the maximum intensity, which is 255) at different normalized distances (x/D, where D is tube diameter) from the sonotrode tip (Fig. S3 for A = 60% and Fig. 12 for A = 100%). It is clear that as the distance from the sonotrode increases, the normalized SCL intensity decreases more rapidly at higher flow rates, whereas Q = 0 and 200 ml/min conditions maintain higher relative intensities in the far-field region. This reveals that stagnant or weakly flowing conditions favor the persistence of chemically active cavitation downstream, likely owing to longer bubble residence times in the cavitation zone, which allow bubbles to grow to sizes capable of intense collapse and higher radical production. Also, high flow rates may promote more asymmetrical bubble collapse, leading to reduced collapse temperatures and diminished radical production [48]. Additionally, the higher shear associated with external flow can induce bubble fragmentation into smaller bubbles, and they undergo stable oscillations rather than energetic collapses, limiting reactive radical generation and overall sonochemical activity. [66].

**Fig. 13:**
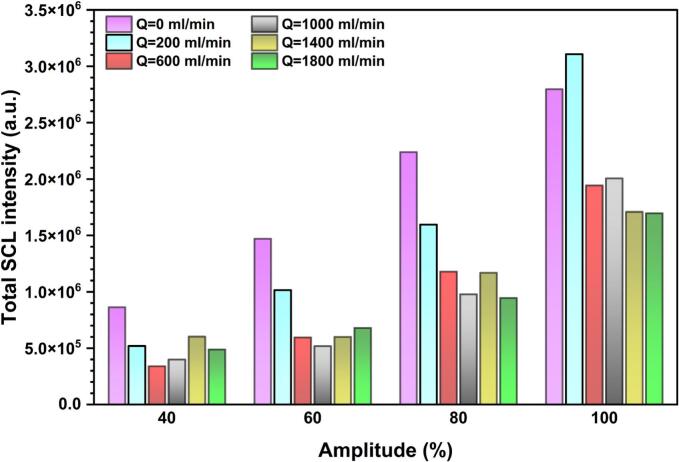
Total SCL intensity under various flow rates and ultrasonic amplitudes in co-current configuration.

The positive effect of stagnant and low-flow conditions is further supported by the analysis of the total SCL intensity as shown in Fig. 13. Since SCL emission intensity is proportional to the number of chemically active bubbles, the total SCL intensity provides a global measure of cavitation-driven chemical activity. At low US amplitude, only the no-flow case exhibits a higher total SCL intensity. Yet, at higher amplitudes, both Q = 0 ml/min and Q = 200 ml/min result in significantly greater total SCL intensities compared with higher flow rates. Also, a notable finding is that a linear relationship between total SCL intensity and ultrasonic amplitude can be observed only under no-flow conditions, whereas this linearity is slightly disrupted under flow conditions.

In ultrasonic systems, it is important to distinguish whether high SCL intensity is limited to a small zone or distributed over a broader zone, as a wider and more uniformly distributed SCL region is commonly preferred for sonochemical reactors [67].Therefore, the SCL area was measured and is presented in Fig. S4. The results show that the higher total SCL intensities discussed above (Fig. 13) are also associated with larger SCL areas, indicating that the enhancement in sonochemical activity is not limited to a confined zone but extends over a wider region. Similarly, the positive effect of increasing amplitude on the SCL area is more pronounced at Q = 0 ml/min and 200 ml/min.

**Fig. 14:**
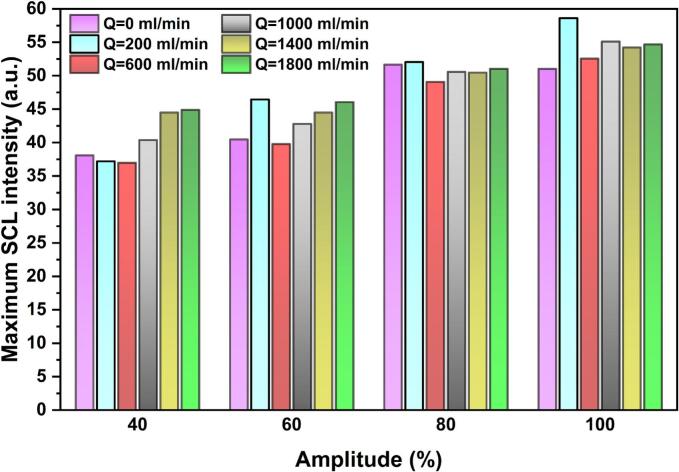
Maximum SCL intensity under various flow rates and ultrasonic amplitudes in co-current configuration.

In both shadowgraphy and SCL images, the highest bubble population density and the peak chemical activity are consistently observed at the sonotrode tip. Because the activity of this region is critical for sonochemical reactions, the maximum SCL intensity values were extracted for each scenario and are given in Fig. 14 as an indicator of the hotspot strength at the acoustic source. In contrast to the total SCL intensity and SCL area, the maximum SCL intensity shows a relatively weak dependence on both ultrasonic amplitude and flow rate. This suggests that, within the investigated operating window, variations in flow rate or amplitude do not substantially modify the severity of chemical activity in the immediate vicinity of the sonotrode tip, but instead primarily affect the spatial distribution of sonochemical activity.

#### 3.3.2 Counter-current configuration

**Fig. 15:**
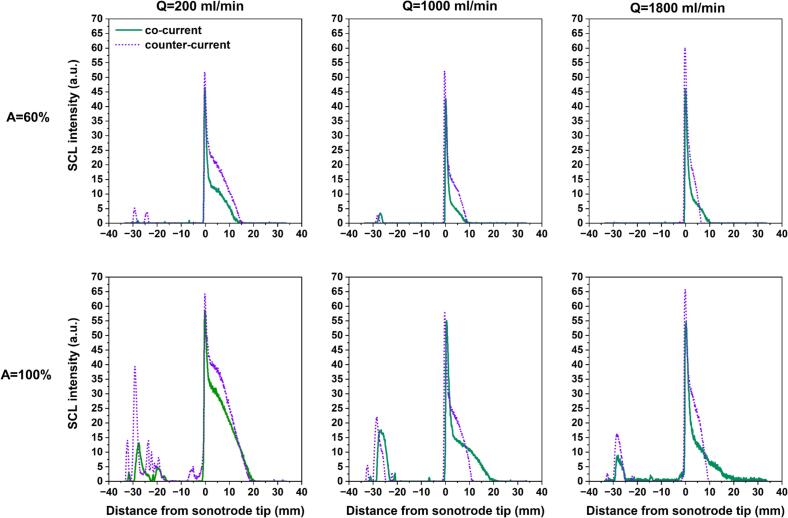
Comparison of SCL intensity profiles along the tube length obtained with co-current and counter-current operation.

SCL imaging in counter-current operation was performed at Q = 200, 1000, and 1800 ml/min and US amplitudes of 60 and 100%. Fig. S5 presents the SCL images in counter-current mode, and Fig. 15 compares the SCL intensity profiles along the length of the tube between co-current and counter-current configurations. As can be seen, counter-current consistently shows a greater peak SCL emission at the sonotrode tip compared with co-current mode, which is also confirmed through comparison of maximum SCL intensities in Fig. S6. Moreover, the SCL intensity in counter-current decays more gradually in the near-field region, revealing enhanced sonochemical activity close to the sonotrode. At the low flow rate of 200 ml/min, where the external fluid flow weakly opposes the acoustic streaming, both co-current and counter-current modes exhibit a similar downstream penetration of chemical activity (locations where the SCL intensity becomes zero) for both tested amplitudes. Nevertheless, as the opposing flow rate increases, the downstream extent of the SCL zone shifts backward (closer to the sonotrode tip), indicating that stronger counter-current flow can push the chemically active region upstream.

**Fig. 16:**
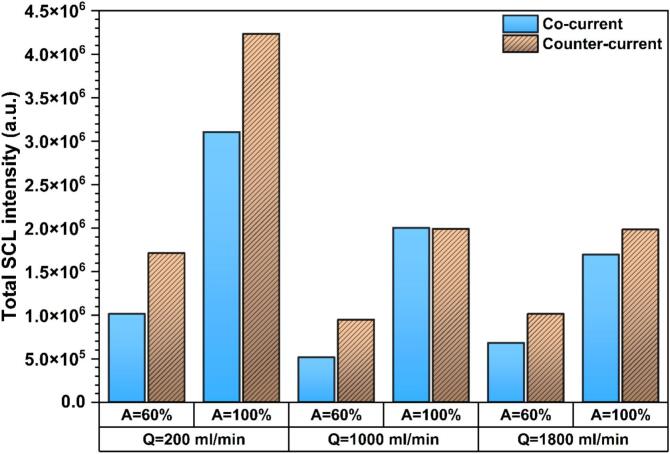
Comparison of total SCL intensity between co-current and counter-current flows at Q = 200, 1000, and 1800 ml/min, and set amplitude of 60 and 100%.

Fig. 16 and S7 respectively present the total SCL intensity and SCL area under counter-current mode together with their co-current counterparts. Similar to co-current flow, the low flow rate of 200 ml/min is the most favorable condition for achieving both higher overall sonochemical activity and larger luminescence areas under counter-current operation, which is clearly linked to enhanced bubble activity and radical yield. Although in some cases the counter-current SCL profiles become lower than those of co-current in the far-field region (Fig. 15), the total SCL intensity is higher under counter-current conditions for most cases, except for Q = 1000 ml/min at A = 100%, where both modes yield similar intensity. In addition, the SCL area generated under counter-current operation is significantly larger than that of co-current, showing the improved spatial distribution of chemically active cavitation.

**Fig. 17:**
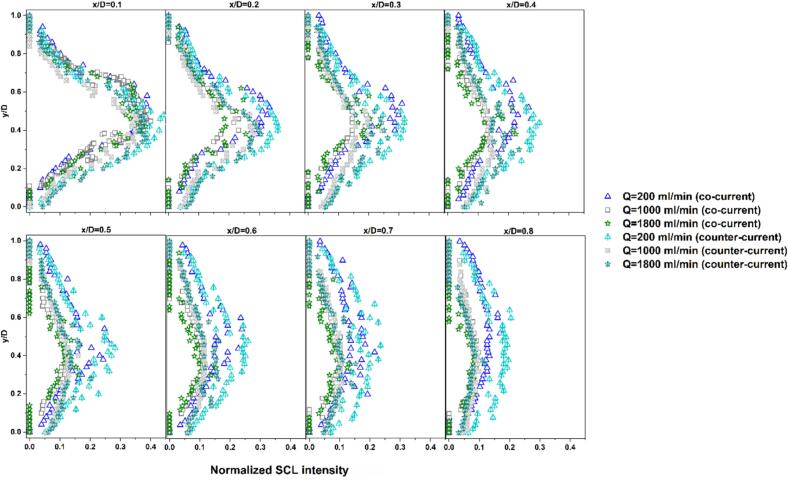
Comparison of normalized radial SCL intensity profiles at different normalized distances from sonotrode tip (x/D) under flow rates of 200, 1000, and 1800 ml/min and set amplitude of 100% with co-current and counter-current operation.

In order to get more insight into the effect of fluid flow direction, the normalized SCL intensity at different axial positions (x/D) was plotted and compared between co-current and counter modes (Fig. S8 for A = 60% and Fig. 17 for A = 100%). It is evident that, in the near-field zones, particularly up to $\frac{x}{D}=$ 0.4, counter-current operation exhibits not only higher SCL intensity but also a wider radial distribution, with bright regions extending closer to the tube walls. This implies that counter-current configuration enhances both the intensity and the spatial spread of sonochemical activity in the near-field region. The observed asymmetry in luminescence intensity, especially in the far-field, is attributed to the asymmetrical configuration of the tube inlet/outlet port.

From a mechanistic perspective, when the bulk flow and acoustic streaming are co-current, cavitation bubbles are more likely to be swept out of the high-pressure acoustic region faster, and they would therefore undergo fewer oscillation cycles available for bubble growth and intense implosion. On the other hand, counter-current flow partially counteracts this drift, increasing the likelihood of local stagnation regions and/or vortices arising from acoustic–hydrodynamic interactions [40]. As illustrated in Fig. S9, which presents the hydrodynamic behavior for Q = 1000 ml/min and A = 100%, the counter-current mode generates a weaker and more confined jet, thereby increasing the effective residence time within the active region. Consequently, this altered hydrodynamic environment extends bubble residence within the sonication zone, enhancing the probability for bubbles to reach sizes conducive to more violent collapses, ultimately resulting in brighter and more spatially distributed SCL emission.

Comparison of the SCL and shadowgraphy results demonstrates that, in acoustic cavitation reactors, not all the triggered cavitation bubbles are chemically active. While both techniques consistently show that the counter-current configuration generally promotes both larger cavitation clouds and enhanced sonochemical activity, and that low/moderate flow rates are favorable for both cavitation development and chemical activity, a notable discrepancy is observed under stagnant conditions. In this case, the two techniques yield notably different trends, suggesting that the mere presence of cavitation bubbles or clusters does not necessarily guarantee effective radical production. This behavior suggests that under certain conditions, cavitation bubbles may exist but undergo collapses with insufficient intensity to generate reactive radical species, highlighting the importance of distinguishing between physical cavitation occurrence and chemical cavitation activity.

### 3.4 KI dosimetry and sonochemical efficiency

In sonochemical reactors, apart from mapping the chemically active zone, it is important to quantify the global produced reactive radicals in the system, as it directly dictates the chemical effects of cavitation, such as the degradation of organic pollutants. Although larger SCL areas and intensities are often indicative of enhanced local radical generation, this must be validated through direct radical quantification using chemical dosimetry techniques. Hence, the KI dosimetry experiments were performed under flow conditions from 200 to 1800 ml/min and US amplitudes of 60% and 100% for the co-current, and 100% for the counter-current mode. It should be noted that, although quantifying radical production under the no-flow case would be of interest, the experiments could not be performed because the tubular reactor was not equipped with a sampling port. Therefore, KI dosimetry measurements were only limited to flow conditions.

**Fig. 18:**
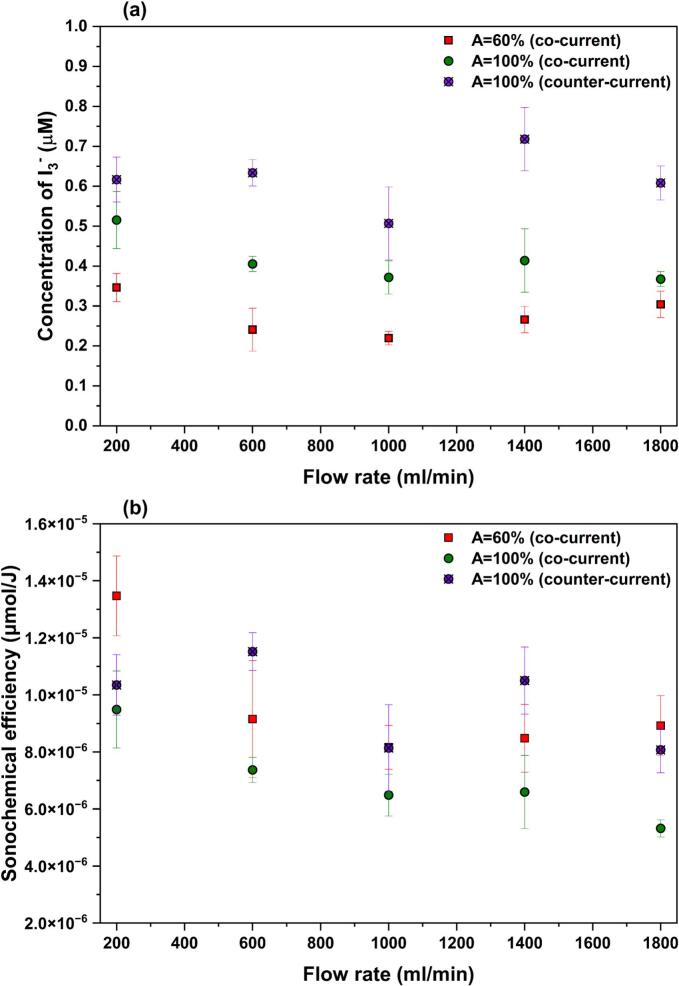
(a) KI dosimetry results and (b) corresponding sonochemical efficiency under different flow rates, US amplitudes of 60% and 100% for co-current mode, and US amplitude of 100% for counter-current mode.

Fig. 18(a) presents the triiodide (I_3_^−^) concentration as a function of flow rate. As shown, in co-current mode, increasing the amplitude from 60% to 100% results in a higher amount of radical production across all the flow rates. For the flow rates between 200 and 1400 ml/min, increasing the amplitude leads to about 1.5–1.7 times greater radical production, which is owing to the higher number and size of the cavitation bubbles [68], as well as the elongation of bubbly zones, as confirmed by shadowgraphy. However, at the highest flow rate (1800 ml/min), the enhancement due to increased amplitude is reduced to about 1.2 times, indicating that at very high flow rates the positive effect of amplitude on radical production becomes less pronounced. This trend is consistent with the SCL intensity profiles (Fig. 12), where the difference between A = 60% and 100% is more evident at lower flow rates and diminishes at Q = 1800 ml/min.

The influence of flow rate on radical production under co-current flow shows that, at both amplitudes, the lowest investigated flow rate (Q = 200 ml/min) yields the highest radical concentration. Increasing the flow rate beyond this value leads to a slight decrease in radical production, followed by a relatively weak dependence on flow rate at higher values, with nearly constant radical levels at A = 100% and a marginal increase at A = 60%. As mentioned earlier, the KI dosimetry experiments were conducted under recirculation (closed-loop) mode. For a fixed processing time (5 min), increasing the flow rate increases the number of recirculation (from 7 passes at Q = 200 ml/min to 63 passes at Q = 1800 ml/min), while simultaneously reducing the residence time of the liquid within the cavitation zone during each pass. As a result, the total exposure time to ultrasound remains independent of flow rate. Instead, radical production is determined by (i) whether flow-induced acoustic streaming modifies bubble retention within the cavitation zone and (ii) the intrinsic chemical activity of the cavitation bubbles under different flow conditions. The former requires a specific hydrodynamic study and will be addressed in future research; the latter is supported by the SCL results, which show that Q = 200 ml/min induces a larger and more intense chemically active zone. Consequently, the higher radical production observed at this flow rate is primarily attributed to the intensified chemical activity of cavitation bubbles as confirmed with SCL mapping.

Fig. 18(a) also illustrates that switching the flow configuration from co-current to counter-current at A = 100% gives rise to a significant increase in radical production. This observation is consistent with the SCL results, which show that counter-current generally enhances sonochemical activity compared with co-current flow. However, according to the SCL results, in which Q = 200 ml/min exhibits the highest SCL intensity among the flow conditions, the KI dosimetry results indicate that the radical concentration at Q = 1400 ml/min is slightly higher than other conditions, and at Q = 200, 600, and 1800 ml/min the concentration is nearly constant. This discrepancy can be justified by considering the fundamental differences between these two techniques. In KI dosimetry, radical detection depends on the ability of reactive radicals, primarily generated at or near the bubble–liquid interface, to escape into the bulk liquid and react with I^−^ ions. The timescale for radical diffusion into the bulk is very short (in the order of 10^−9^–10^−7^ s), and radicals that fail to penetrate the surrounding liquid might undergo rapid recombination [69]. In contrast, SCL emission arises from local reactions occurring in the immediate vicinity of bubble collapse, and therefore does not require long transport of radicals into the bulk solution. Consequently, even radicals that do not fully diffuse away from the interface can still contribute to SCL emission. As a result, local cavitation intensity and bulk radical yield may respond differently based on the hydrodynamics around the bubble, particularly under counter-current flow where strong shear, recirculation, and bubble deformation can alter radical transport efficiency. This difference between SCL and KI dosimetry results in the continuous ultrasonic reactor has also been observed previously [37]. From a practical standpoint, the most relevant metric in sonochemistry is the total amount of reactive species that effectively survive and participate in chemical transformations, i.e., radicals that escape recombination within or at the bubble–liquid interface. Therefore, KI dosimetry provides a more representative measure of the effective radical yield, as it quantifies iodine formation resulting from radicals that have diffused into the bulk liquid and contributed to chemical reactions. However, SCL mainly reflects the intensity of radical formation events occurring inside or very close to the collapsing bubbles.

The corresponding sonochemical efficiency is shown in Fig. 18(b). In co-current mode, the sonochemical efficiency at A = 100% is notably lower than that at A = 60%, showing that although higher amplitude produces a higher quantity of radicals, this increase is not proportional to the acoustic energy delivered to the system. Under co-current operation, higher ultrasonic amplitude generates stronger acoustic streaming in front of the sonotrode. Although operation at 100% amplitude leads to a larger cavitation cloud area than at 60%, the intensified acoustic streaming at 100% amplitude can transport bubbles out of the cavitation zone more rapidly, thereby reducing their effective exposure time in the active zone.

In addition, although increasing the flow rate slightly enhances the calorimetric power dissipated into the liquid, this additional energy does not translate into a proportional increase in radical production. As a result, the Q = 200 ml/min yields the highest sonochemical efficiency for both 60% and 100% amplitudes. This shows that, at higher flow rates and amplitudes, a larger fraction of the supplied acoustic energy is dissipated as heat rather than contributing to sonochemical reactions. This behavior agrees with the concept that not all cavitation bubbles contribute equally to sonochemistry. At high amplitudes and strong hydrodynamic transport, a larger population of bubbles may oscillate inertially or collapse asymmetrically without reaching the extreme conditions required for efficient radical formation. These bubbles still contribute to energy dissipation through viscous losses, shock waves, and liquid heating, but remain chemically inefficient. As a result, the system exhibits slightly higher calorimetric power but reduced sonochemical efficiency, which is in agreement with earlier interpretations distinguishing between dissipated energy density and chemically active cavitation bubbles [56].

Under counter-current operation at A = 100%, the sonochemical efficiency is observed to be greater than that of co-current flow, showing that counter-current flow not only promotes radical production but also improves the effectiveness of energy conversion into sonochemical activity. However, the sonochemical efficiency under counter-current conditions shows a non-monotonic dependency on flow rate, indicating a more complex interplay between hydrodynamics and sonochemistry in counter-current mode.

It is also important to note that the present sonoreactor was operated at a low US frequency of 35 kHz, and the observations reported here are limited to the the interaction between fluid flow and sonochemical activity under low-frequency ultrasonic irradiation. This interplay may differ at higher frequencies (>100 kHz), where bubble dynamics and cavitation activity are known to change significantly. Consequently, the conclusions drawn regarding the influence of flow rate on radical generation are primarily applicable to low-frequency sonoreactor systems.

## 4 Conclusion

This study experimentally investigated the influence of fluid flow rate and ultrasonic amplitude on sonochemical activity and cavitation cloud under co-current and counter-current configurations in a 35 kHz flow-through tubular sonoreactor using a combination of characterization techniques. The results showed a linear relationship between calorimetric power and ultrasonic amplitude under the studied flow rates (0–1800 ml/min). Although higher flow rates slightly increased calorimetric power, ultrasonic efficiency remained nearly constant due to increased electrical input required to maintain oscillation amplitude. Counter-current operation also yielded slightly higher calorimetric power. Shadowgraphy revealed that cavitation clouds elongated with increasing flow rate up to 600 ml/min, beyond which cavitation extent decreased, and it was confirmed by gray intensity analysis and cloud area measurements. SCL results showed that stagnant fluid and a low flow rate of 200 mL/min were most favorable for sonochemical activity, yielding higher intensities, larger reactive zones, and greater axial penetration. These effects are attributed to longer bubble residence times and reduced disruption of bubble dynamics. Counter-current flow consistently enhanced SCL intensity and area compared to co-current operation, despite shifting the active zone closer to the sonotrode at higher flow rates. KI dosimetry results largely corroborated the SCL trends, indicating greater radical production at higher amplitudes and lower flow rates, while also revealing higher sonochemical efficiency at lower amplitudes. Overall, the results demonstrate that not all cavitation bubbles contribute to sonochemistry, emphasizing the need to distinguish between cavitation presence and chemical effectiveness in flow-through ultrasonic reactors.

## CRediT authorship contribution statement

**Amirmohammad Javidani:** Writing – original draft, Visualization, Validation, Methodology, Investigation, Formal analysis, Data curation, Conceptualization. **Martine Poux:** Writing – review & editing, Validation, Supervision, Funding acquisition, Conceptualization. **Joelle Aubin:** Writing – review & editing, Validation, Supervision, Conceptualization. **Hélène Chaumat:** Writing – review & editing, Validation, Supervision, Conceptualization. **Laurie Barthe:** Writing – review & editing, Visualization, Supervision, Project administration, Conceptualization.

## Declaration of competing interest

The authors declare that they have no known competing financial interests or personal relationships that could have appeared to influence the work reported in this paper.
