# Supplementary material for: Revealing the interplay between flow dynamics and cavitation activity in a flow-through sonoreactor
Source: Ultrason Sonochem. 2026 Apr 15;129:107854. doi: 10.1016/j.ultsonch.2026.107854 (PMC13101780; doi:10.1016/j.ultsonch.2026.107854)
Supplement: Supplementary Data 1 [file mmc1.docx]

**Supporting material**

**Revealing the interplay between flow dynamics and cavitation activity in a flow-through sonoreactor**

**Shadowgraphy imaging**

In AC shadowgraphy, cavities appear as dark regions (low gray intensity) relative to the surrounding liquid phase, which appears brighter (high gray intensity). For ease of analysis and visualization, the grayscale images were inverted such that higher gray intensities correspond to a higher density of the cavitation bubbles, and lower intensities represent the liquid-dominated regions. The increased grayscale intensity after inversion is directly linked to the local number and size of the cavitation bubbles. For improved visualization, the processed images were then converted to a rainbow colormap, allowing clearer discrimination between highly dense cavitation cloud, less dense cavitation regions, and isolated cavitation clusters. Quantitative image analysis was performed along the axial direction from the sonotrode tip, corresponding to the direction of ultrasound propagation. A minimum gray intensity threshold of 40 was selected based on visual inspection and noise suppression. Hence, values in the range of 40 – 255 were assumed to correspond to active cavitation zones. Also, the highly dense cavitation cloud was identified within the gray intensity range of 80 – 255, which corresponds to the boundary of the conical shape of dense cavitation zone. By binarizing the images within this range, the area of the dense cavitation zone was obtained.

**Sonochemiluminescence**

In an 8-bit image, the grayscale intensity ($I$) is represented by 256 discrete levels (0 – 255). Accordingly, the intensity of the emitted blue light in SCL images is quantified by the corresponding gray intensity. The total SCL intensity (in arbitrary unit, a.u.), which is an indicator of cavitation chemical activity, within a defined rectangular region of interest (ROI) was calculated as the sum of the gray intensities of all pixels inside the ROI, as given in Eq. (1). The ROI had a fixed height equal to the inner diameter of the tube (250 pixels or 1 cm) and a fixed width of 1680 pixels (6.7 cm), extending axially from the fluid inlet/outlet port (co-current or counter-current mode) to the downstream region where no detectable blue-light emission was observed.

| ${SCL}_{total}=\sum_{x=1}^{W} \sum_{y=1}^{H} I(x,y)$ | (1) |
| --- | --- |

In sonoreactors, a broader spatial distribution of SCL activity within is more favorable. Therefore, in addition to the total SCL intensity, the SCL area was also determined by applying an intensity threshold between 20 to 255 for noise removal, and the boundaries within this rangecorresponds to the SCL area.

**
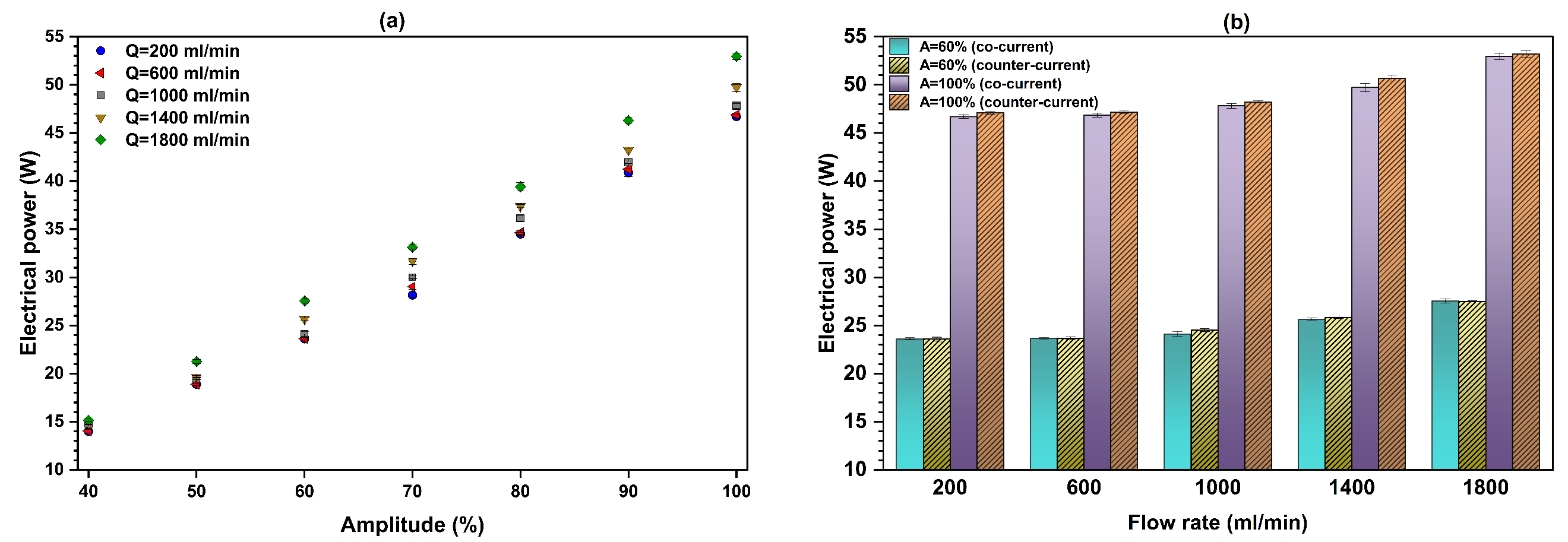
**

**Fig. S1.** (a) Effect of external flow rate on ultrasonic electrical power under different amplitudes and co-current mode, (b) comparison of electrical power between co-current and counter-current configurations working at flow rates ranging from 200 to 1800 ml/min and US amplitudes of 60 and 100%.


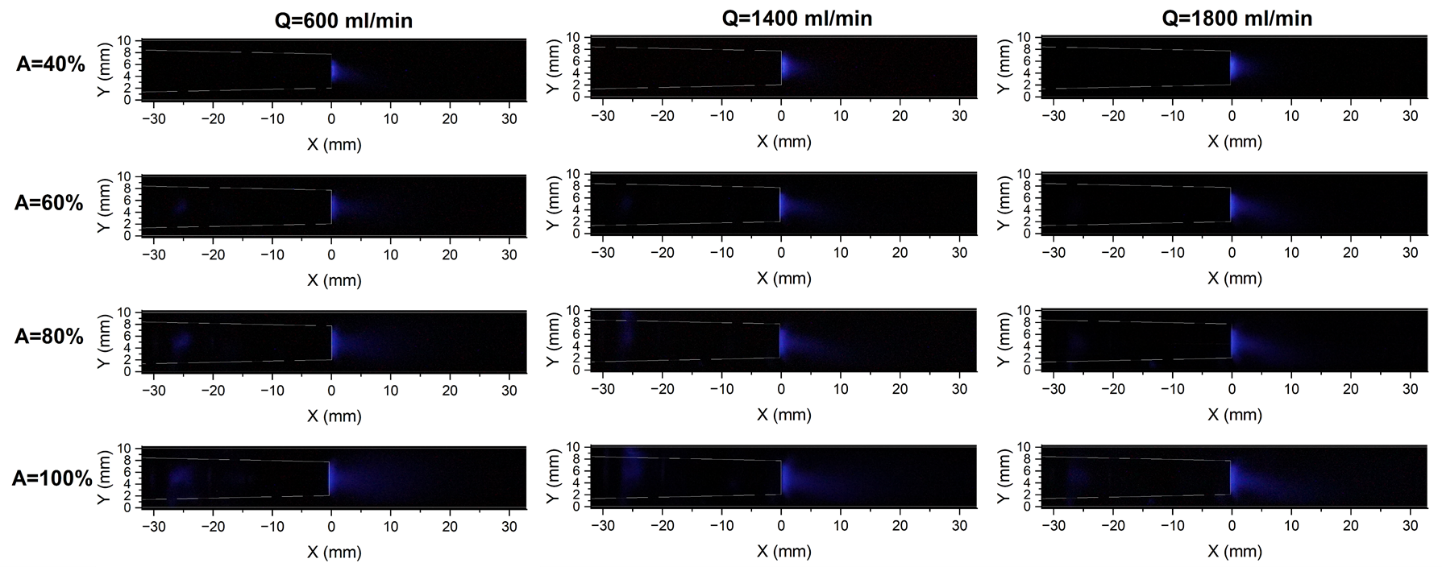


**Fig. S2.** SCL images obtained at Q=600, 1400, and 1800 ml/min and varying amplitudes from 40 to 100% in co-current mode.


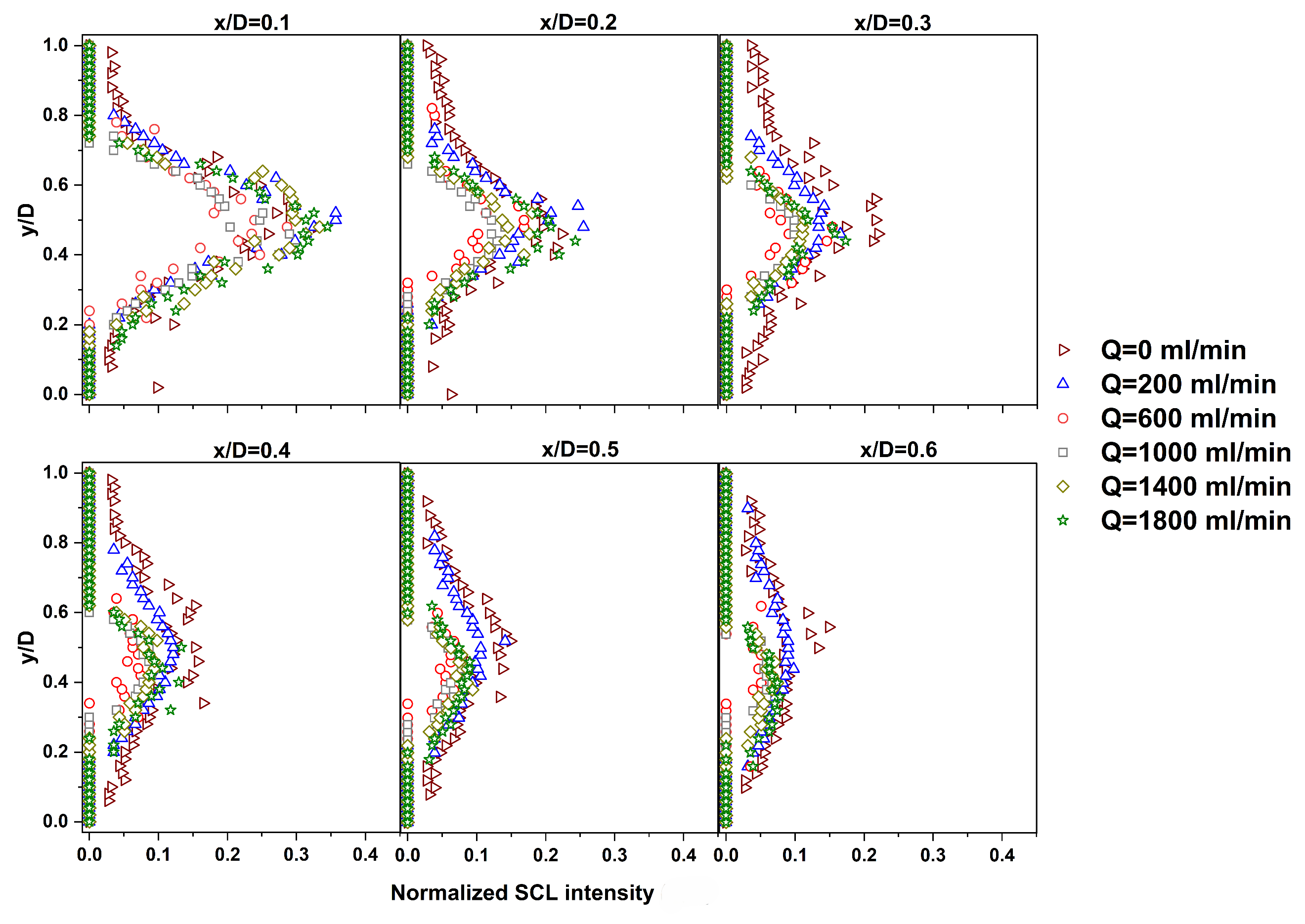


**Fig. S3.** Normalized SCL intensity at different normalized distances from sonotrode tip (x/D) for varying flow rates and A=60% working under co-current flow.

**
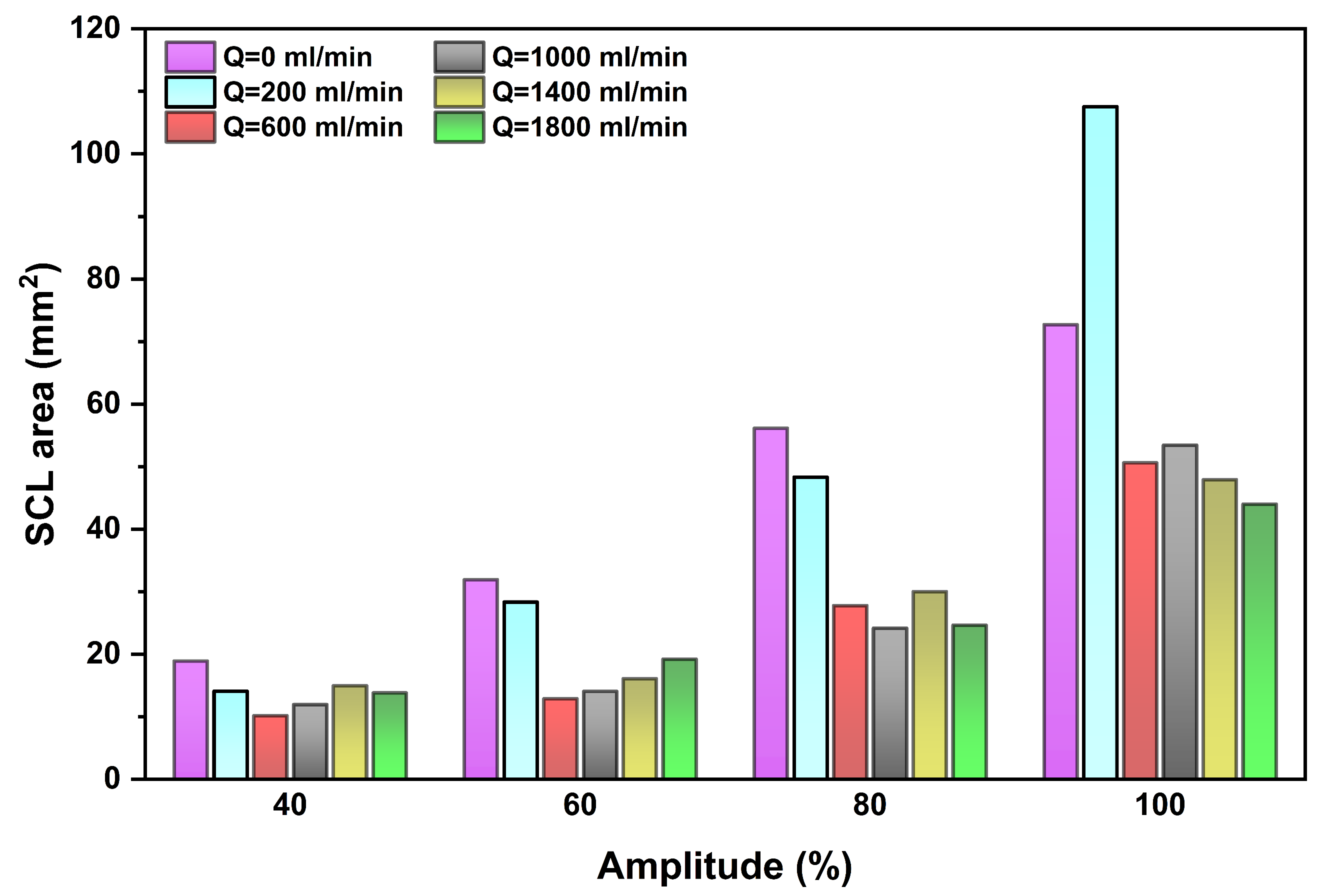
**

**Fig. S4.** SCL area generated at varying flow rates (0 – 1800 ml/min) and US amplitudes (40 – 100%) in co-current configuration.


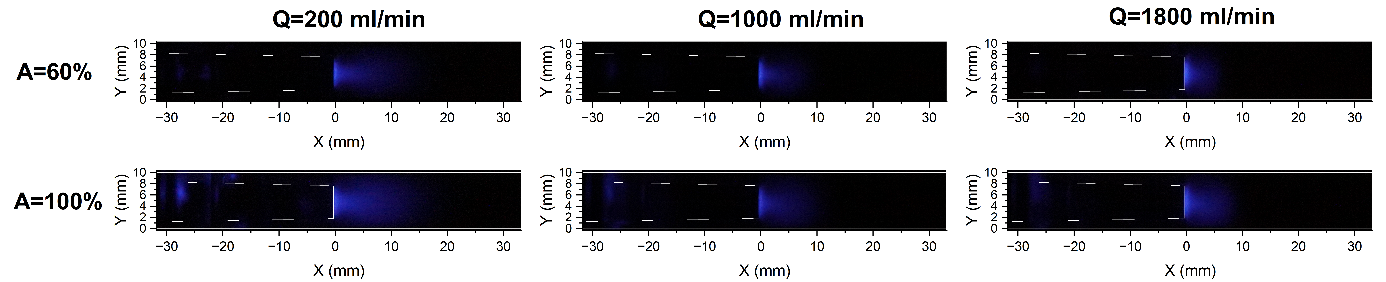


**Fig. S5**. SCL images acquired at flow rates of 200, 1000, and 1800 ml/min and amplitudes of 60 and 100% under counter-current mode.


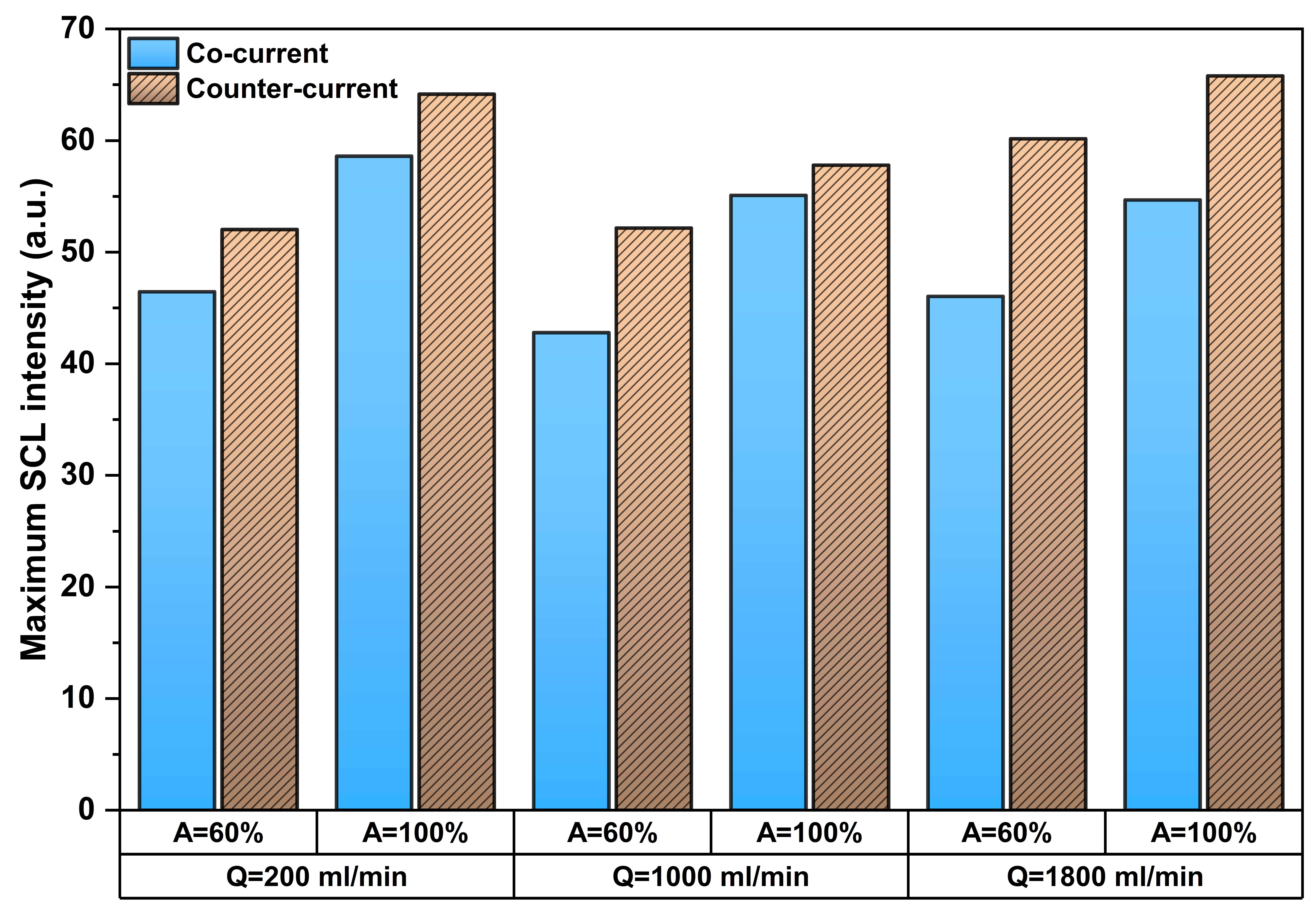


**Fig. S6.** Comparison of maximum SCL intensity between co-current and counter-current modes at different flow rates and amplitudes.

**
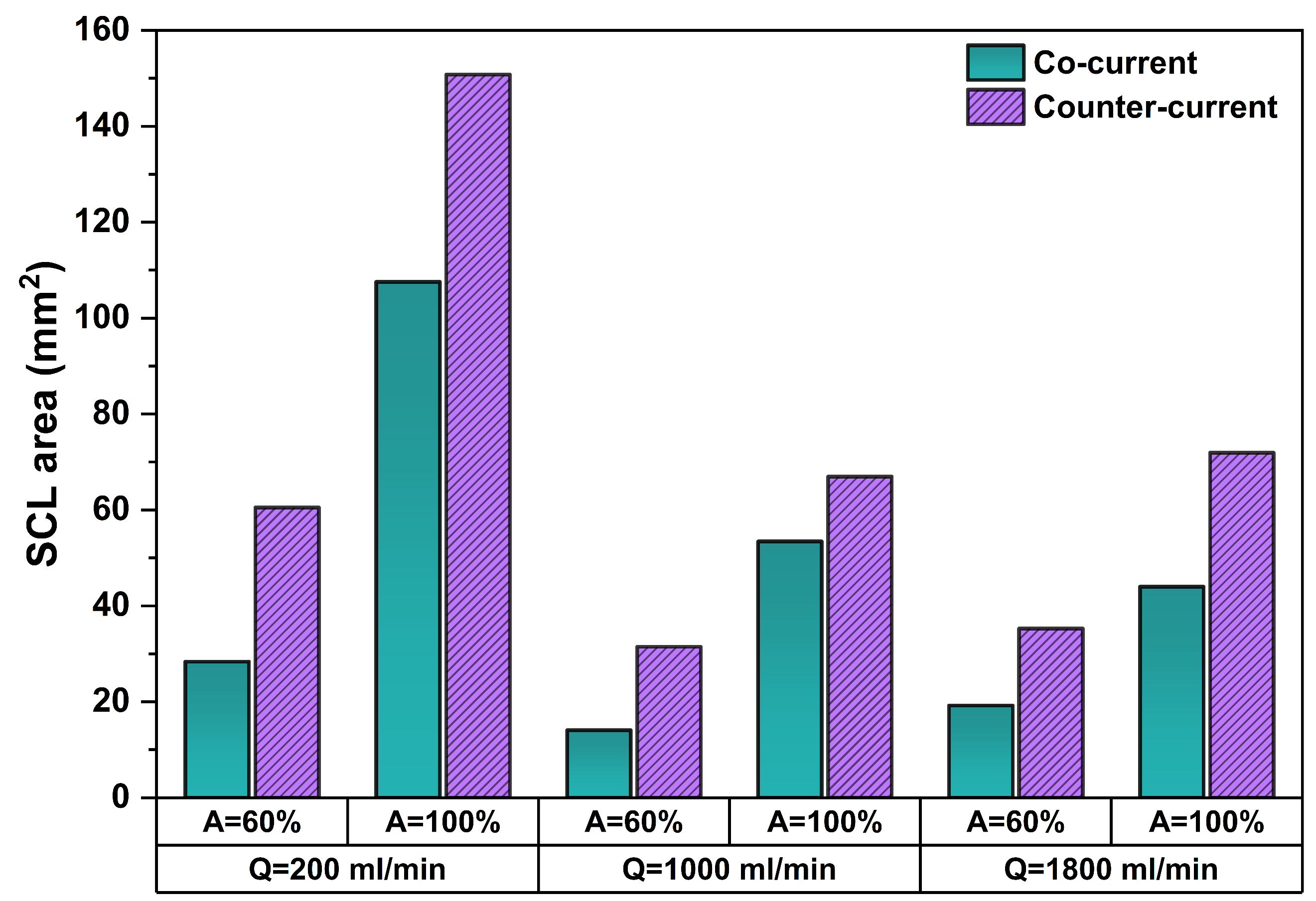
**

**Fig. S7.** Comparison of SCL area between co-current and counter-current modes at different flow rates and amplitudes.


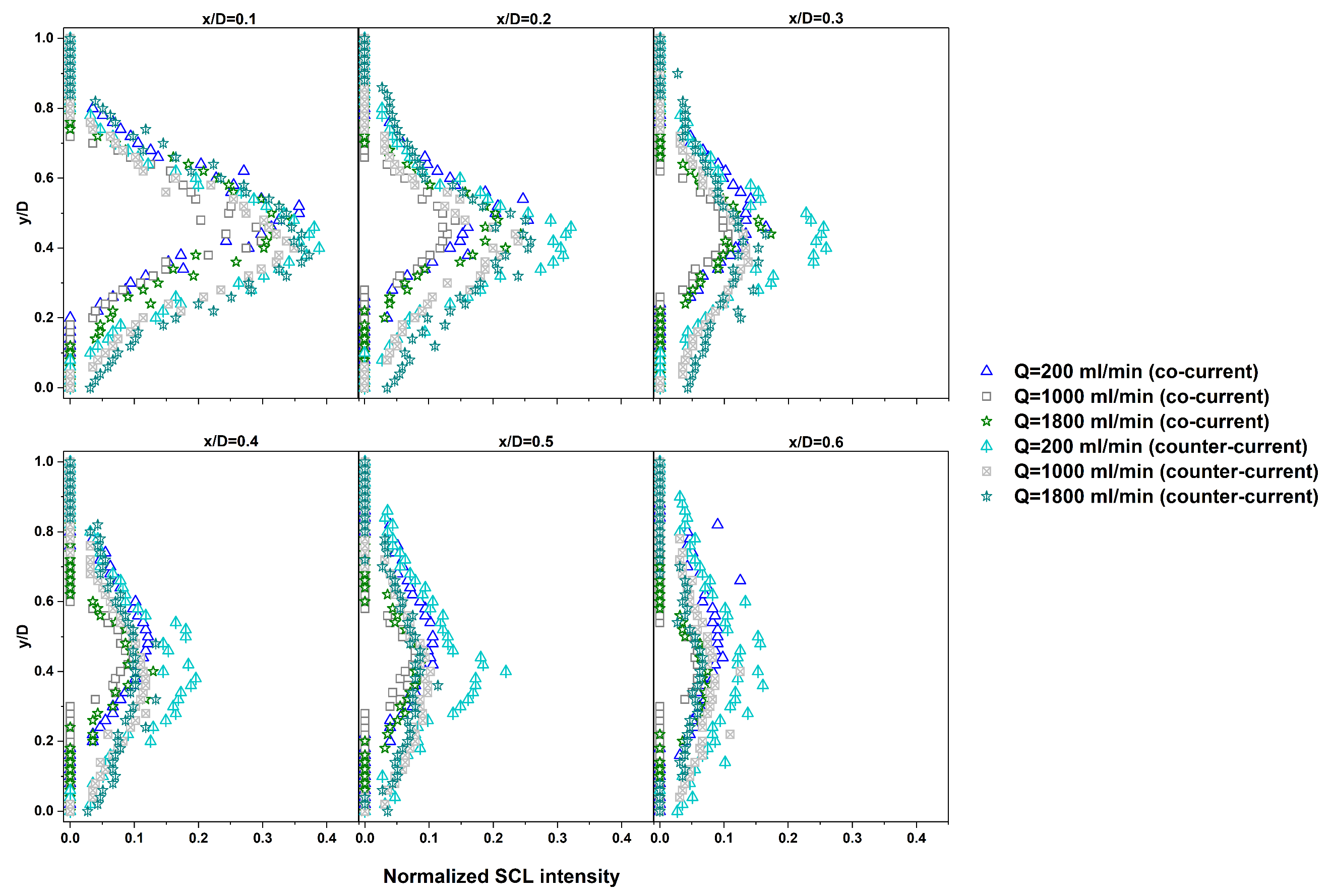


**Fig. S8.** Comparison of normalized SCL intensity between co-current and counter-current at different normalized distances from sonotrode tip (x/D) under flow rates of 200, 1000, and 1800 ml/min and set amplitude of 60%.


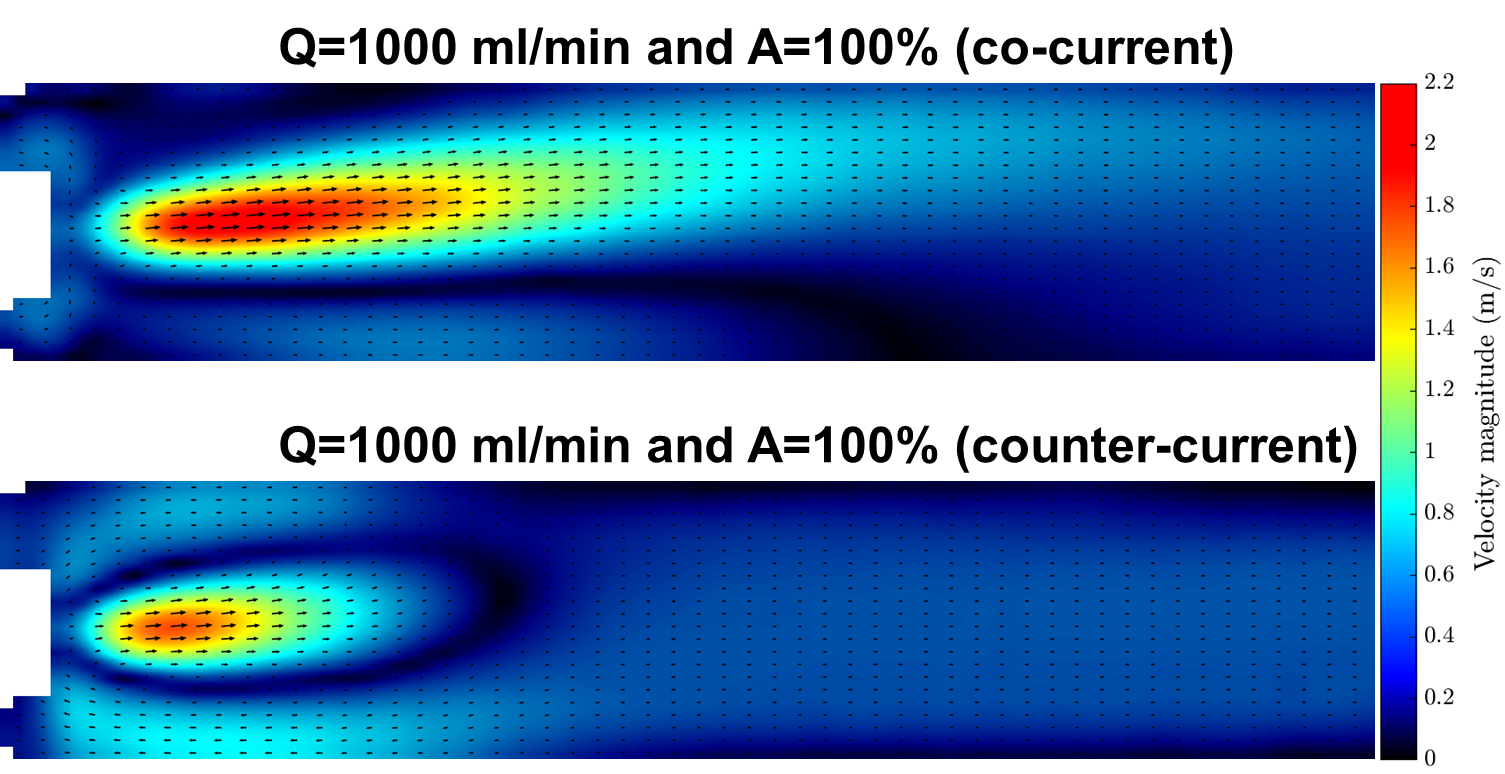


**Fig. S9.** Comparison of co-current and counter-current time-averaged velocity profile at Q=1000 ml/min and A=100%.
